# Quantitative EEG analysis in typical absence seizures: unveiling spectral dynamics and entropy patterns

**DOI:** 10.3389/fnhum.2023.1274834

**Published:** 2023-10-17

**Authors:** Alioth Guerrero-Aranda, Evelin Ramírez-Ponce, Oscar Ramos-Quezada, Omar Paredes, Erick Guzmán-Quezada, Alejandra Genel-Espinoza, Rebeca Romo-Vazquez, Hugo Vélez-Pérez

**Affiliations:** ^1^Depto. de Ciencias de la Salud, Centro Universitario de Los Valles, Universidad de Guadalajara, Guadalajara, Jalisco, Mexico; ^2^Clínica de Epilepsia, Hospital “Country 2000, ” Guadalajara, Jalisco, Mexico; ^3^Depto. de Bioingeniería Traslacional, Centro Universitario de Ciencias Exactas e Ingenierías, Universidad de Guadalajara, Guadalajara, Jalisco, Mexico; ^4^Mecatrónica, Instituto Tecnológico y de Estudios Superiores de Monterrey, Escuela de Ingenierías y Ciencias (ITESM) Campus Guadalajara, Zapopan, Mexico; ^5^Depto. de Ciencias Computacionales, Centro Universitario de Ciencias Exactas e Ingenierías, Universidad de Guadalajara, Guadalajara, Jalisco, Mexico; ^6^Depto. de Electromecánica, Universidad Autónoma de Guadalajara, Zapopan, Jalisco, Mexico; ^7^Centro Infantil para el Desarrollo Neuroconductual, Hermosillo, Sonora, Mexico

**Keywords:** EEG, entropy, epilepsy, ictal, interictal, spectral analysis, spike-and-wave, typical absence seizure

## Abstract

A typical absence seizure is a generalized epileptic event characterized by a sudden, brief alteration of consciousness that serves as a hallmark for various generalized epilepsy syndromes. Distinguishing between similar interictal and ictal electroencephalographic (EEG) epileptiform patterns poses a challenge. However, quantitative EEG, particularly spectral analysis focused on EEG rhythms, shows potential for differentiation. This study was designed to investigate discernible differences in EEG spectral dynamics and entropy patterns during the pre-ictal and post-ictal periods compared to the interictal state. We analyzed 20 EEG ictal patterns from 11 patients with confirmed typical absence seizures, and assessed recordings made during the pre-ictal, post-ictal, and interictal intervals. Power spectral density (PSD) was used for the quantitative analysis that focused on the *delta, theta, alpha*, and *beta* bands. In addition, we measured EEG signal regularity using approximate (ApEn) and multi-scale sample entropy (MSE). Findings demonstrate a significant increase in *delta* and *theta* power in the pre-ictal and post-ictal intervals compared to the interictal interval, especially in the posterior brain region. We also observed a notable decrease in entropy in the pre-ictal and post-ictal intervals, with a more pronounced effect in anterior brain regions. These results provide valuable information that can potentially aid in differentiating epileptiform patterns in typical absence seizures. The implications of our findings are promising for precision medicine approaches to epilepsy diagnoses and patient management. In conclusion, our quantitative analysis of EEG data suggests that PSD and entropy measures hold promise as potential biomarkers for distinguishing ictal from interictal epileptiform patterns in patients with confirmed or suspected typical absence seizures.

## 1. Introduction

A typical absence seizure is a generalized epileptic event characterized by a sudden, brief alteration of consciousness (Bancaud et al., 1981). During these seizures, individuals often experience loss of awareness and may struggle to recall events that occurred during the episode (Fisher et al., 2017). In-depth semiological analysis can reveal subtle clonic movements that affect various facial regions (Sadleir et al., 2006). Oral and manual automatisms are frequently observed, and patients may show a tendency to continue a behavior that was occurring before seizure onset (Sadleir et al., 2009). Typical absence seizures are an important hallmark of many generalized epilepsy syndromes. They can be examined effectively through simple visual observation (Hirsch and Panayiotopoulos, 2005). Recently, video-electroencephalographic (video-EEG) monitoring has become a valuable tool for investigating these seizures, but it may not be readily available in resource-limited regions where EEG procedures often do not include video acquisition. Consequently, if the technician fails to capture the event during EEG recording (i.e., by not examining the patient), the only means to confirm the occurrence of an absence seizure is through EEG analysis.

The classic EEG pattern observed during a typical absence seizure is characterized by regular, generalized spike-and-wave activity, with a predominant anterior distribution occurring at a frequency of 3 Hz (Koutroumanidis et al., 2017). In certain cases, slight variations in this pattern may include faster irregular spike-and-wave activity, ranging from 3.5–6 Hz, or polyspike-and-wave complexes (Panayiotopoulos et al., 1989). It is important to note, however, that patients may also exhibit interictal epileptiform discharges that share similar features with the ictal pattern, making differentiation challenging (Antwi et al., 2019; Springer et al., 2022). Some features may provide clues to the ictal nature of the pattern, including longer duration, greater frequency, morphology, or evolution, as well as the presence of movement artifacts (e.g., blinking) that may be indicative of an ictal pattern (Koutroumanidis et al., 2017).

Differentiating between ictal and interictal EEG patterns in patients with suspected typical absence seizures is crucial for two main reasons. First, accurate diagnoses are essential for patients with suspected epilepsy. Studies have shown that first-degree healthy relatives of individuals with epilepsy can exhibit interictal epileptiform discharges on their EEGs (genetic traits) (Tashkandi et al., 2019). Therefore, distinguishing these patterns accurately is necessary to avoid misdiagnoses in such cases. Second, differentiating between ictal and interictal patterns is crucial for managing patients with confirmed epilepsy, as the presence of an ictal pattern on EEG recordings may indicate treatment failure and the need to readjust the medication prescribed. Correct identification of these patterns, then, will assist clinicians in making appropriate decisions regarding treatment strategies and optimizing patient care.

However, experience has shown that in some cases, qualitative analysis of EEG recordings through simple visual inspection may not suffice to make definitive determinations (Blumenfeld, 2005). Additional diagnostic techniques and expert interpretation may be necessary to accurately differentiate between these patterns (Trenité and Vermeiren, 2005).

Quantitative EEG analysis (qEEG) involves applying mathematical techniques to time series data to reveal, or extract, information that may not be readily apparent to the human eye. In recent years, qEEG has proven to be a valuable tool in various clinical contexts (Kramer and Kromm, 2019; Koberda, 2021; Tenney et al., 2021; Munjal et al., 2022). One widely used approach in clinical practice consists in analyzing spectral components by means of the classic measures that include absolute and relative power.

Numerous studies in the field of epilepsy research have focused on analyzing the EEG power spectrum (Clemens et al., 2021; Irelli et al., 2022; Zhong et al., 2022). Among these, special attention has been given to the alpha rhythm, which is influenced by cortico-thalamic interactions. These cortico-thalamic interaction have been demonstrated to play a crucial role in generating generalized seizures and spike-and-wave discharges (Bai et al., 2010; Carney et al., 2010).

The hypothesis of this work, thus, concerns the neural dynamics that occur in the moments immediately preceding and following a typical absence seizure. We speculate that these dynamics may induce subtle modifications in background EEG activity, such as slowing, which could potentially be detected in the spectral characteristics of the signals recorded. The study, therefore, analyzes whether discernible differences in the spectral content and the regularity of EEGs during the pre-ictal and post-ictal periods, compared to the interictal state, can serve as indicators to distinguish between the ictal and interictal nature of the epileptiform patterns observed.

## 2. Materials and methods

### 2.1. Participants and EEG recording

Eleven patients (seven females) were recruited from the Epilepsy Clinic at the “Country 2000” Hospital in Jalisco, Mexico, based on the following criteria: (a) age 5–19 years; (b) confirmed diagnosis of either childhood or juvenile absence epilepsy according to the International League Against Epilepsy classification; (c) presence of ictal EEG patterns (3–4 Hz bilateral spike-and-wave discharges) with normal background; and (d) not on anti-seizure medication at the moment of EEG recording. Potential candidates with (a) structural brain abnormalities; (b) neurological or psychiatric disorders; or (c) excessive interference or poor EEG data quality were excluded.

All these patients were referred to the Clinic to undergo an EEG because of clinical events suspecting typical absence seizures. In fact, the EEG led to the confirmation of the diagnosis. That is why all these patients were not on antiseizure medication at the moment of the EEG. Six out of the seven patients demonstrated a previous normal history and just recently had started with these suspicious clinical events. Only one adolescent patient referred a prior bilateral tonic-clonic (BTC) seizure at the age of 11 (4 years before the EEG). We are aware that the potential underreporting or overlooking of typical absence seizures by patients and their caregivers might complicate the precise onset timing of the epilepsy. Regarding the particular case of the adolescent with the previous BTC seizure, there is a chance that the epilepsy might have started at that moment. However, we could not rule out that it was a symptomatic seizure. Not withstanding, we decided to categorize all these patients with new-onset epilepsy, while acknowledging the possibility of delayed recognition.

**Figure 1:**
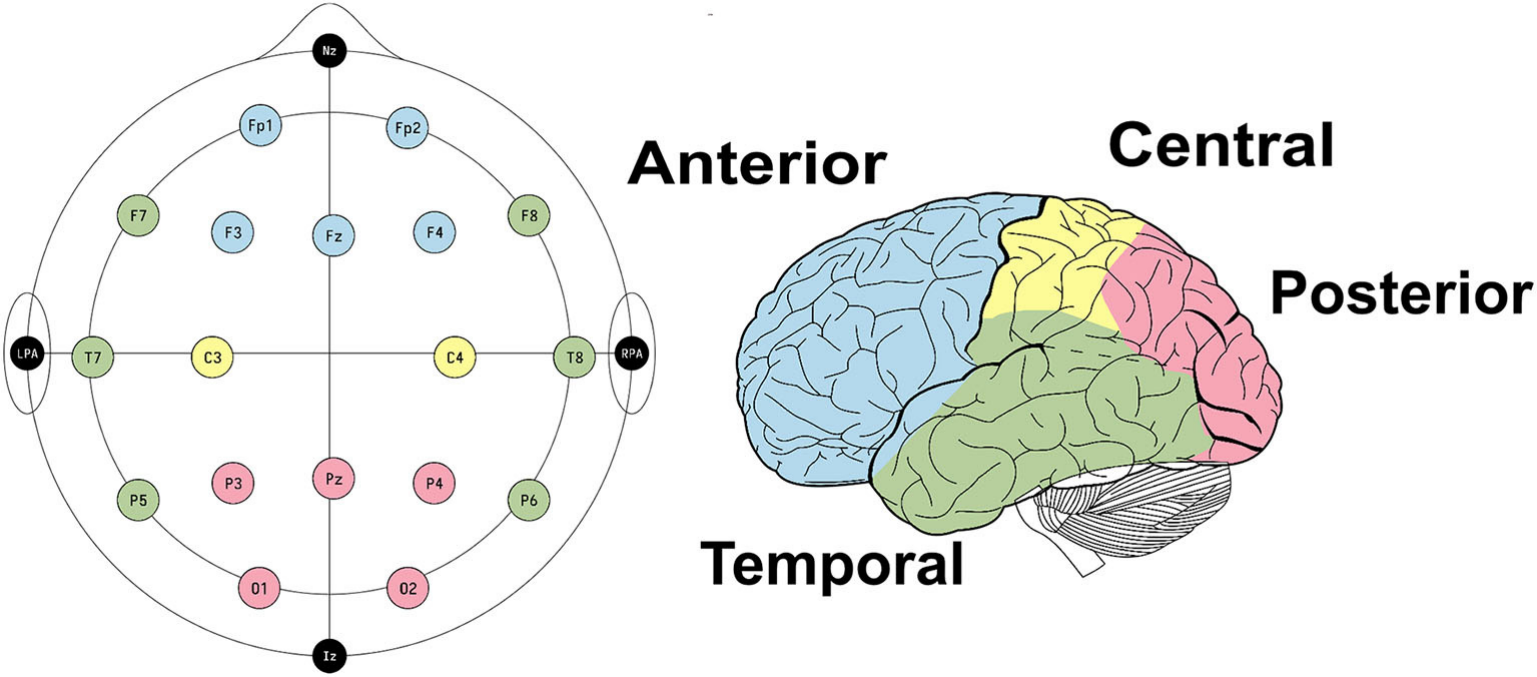
EEG set-up and electrode clusters used to conduct the statistical analysis.

A total of 20 typical absence seizures from these patients were extracted from their EEGs for inclusion in the analysis. All seizures were confirmed through behavioral testing by the EEG technician (online) and analyzed by means of video-EEG by expert clinical epileptologists/neurophysiologists (offline). All EEGs were recorded using 21 electrodes placed according to the International 10–20 System, employing a Cadwell Arc Essentia 32 Channel Clinical EEG system (high-pass filter: 0.53 Hz; low-pass filter: 70 Hz; sampling rate: 256 Hz; <5 kΩ) (Figure 1).

The study protocol was approved by the ethics committee of the “Country 2000” Hospital, and all procedures were conducted following the standards of the Helsinki Declaration. Written informed consent was obtained from each participant (for adults) or her/his parents (for children).

### 2.2. EEG pre-processing

**Figure 2:**
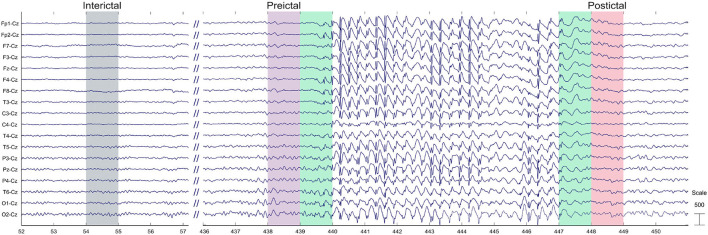
Example of the 1 second windows chosen for the interictal, pre-ictal, and post-ictal states.

In this study, EEG recordings were low-filtered with a cutoff frequency of 55 Hz. Distinct pre-ictal, post-ictal, and interictal segments were considered from each EEG seizure recorded. The pre-ictal segment included a temporal window of 1s prior to the onset of the seizure, while the post-ictal segment comprised the 1s interval following the termination of seizure activity (Figure 2). Not always typical absence seizures start and finish abruptly. Therefore, we decided to rule out 1 second prior to the initial deflection of the first spike of the discharge and 1s after the final deflection of the last wave of the discharge. We then selected for the analysis the 1s windows before and after these previously ruled-out windows and categorized them as pre-ictal and post-ictal states respectively. The temporal markers for both the pre-ictal and post-ictal segments were identified by the medical specialist. The interictal segment was selected while patients were in the eyes-closed condition. All segments were standardized to a duration of 1s to ensure the quasi-stationarity of the EEG signals.

### 2.3. Power spectrum analysis

After pre-processing, the power spectral density (PSD) (Equation 1) of each EEG channel was computed using Fast Fourier Transformation (FFT).


(1) $$\begin{array}{l} PSD\left(f\right)=10*log\left(|\hat{x}\left(f\right){|}^{2}\right) \end{array}$$

A comprehensive assessment of the absolute energy in specific frequency bands—*delta* (1–3 Hz), *theta* (4–7 Hz), *alpha* (8–13 Hz), and *beta* (14–30 Hz)—was conducted. To quantify the energy of each frequency band, we estimated the energy of all frequencies. Considering the factor of inter-subject variability, and in order to facilitate comparisons, all energy values were normalized by dividing each one by the standard deviation of the corresponding frequency band. Finally, the mean energy value for each EEG channel was computed for the interictal, pre-ictal, and post-ictal states for each frequency band.

### 2.4. Entropy

We computed the approximate (ApEn) (Sakkalis et al., 2013) and multi-scale sample entropy (MSE) (Costa et al., 2005) to quantify the regularity of the time series data (Pincus, 1991; Richman and Moorman, 2000). ApEn is used to assess the degree of regularity in the data, with smaller values indicating higher regularity, and larger ones suggesting reduced regularity (Acharya et al., 2012). ApEn can also detect changes in underlying episodic behavior that might not be evident in the data on peak occurrences or amplitudes (Pincus and Keefe, 1992). This tool was also used to compare regularity across the interictal, pre-ictal, and post-ictal states. The computation of ApEn is defined by Equations 2, 3:


(2) $$\begin{array}{l} ApEn={\text{Φ}}_{m}-{\text{Φ}}_{m+1} \end{array}$$

with,


(3) $$\begin{array}{l} {\text{Φ}}_{m}={\left(N-m+1\right)}^{-1}\overset{N-m+1}{\underset{i=1}{\sum }}log\left(N_{i}\right) \end{array}$$

where *N* denotes the number of data points and *m* represents the number of samples where two segments are expected to exhibit similarity. To address variability in the time scales present in the biological signals recorded, we extended the analysis to multi-scale sample entropy, an approach that enhanced the robustness of our quantification of regularity. In summary, our comprehensive data set incorporates various measures, including the energy in four frequency bands, ApEn, and MSE, for all channels in each time window. Each segment in the dataset was labeled to indicate whether it corresponds to an interictal, pre-ictal, or post-ictal state.

### 2.5. Statistical analysis

**Figure 3:**
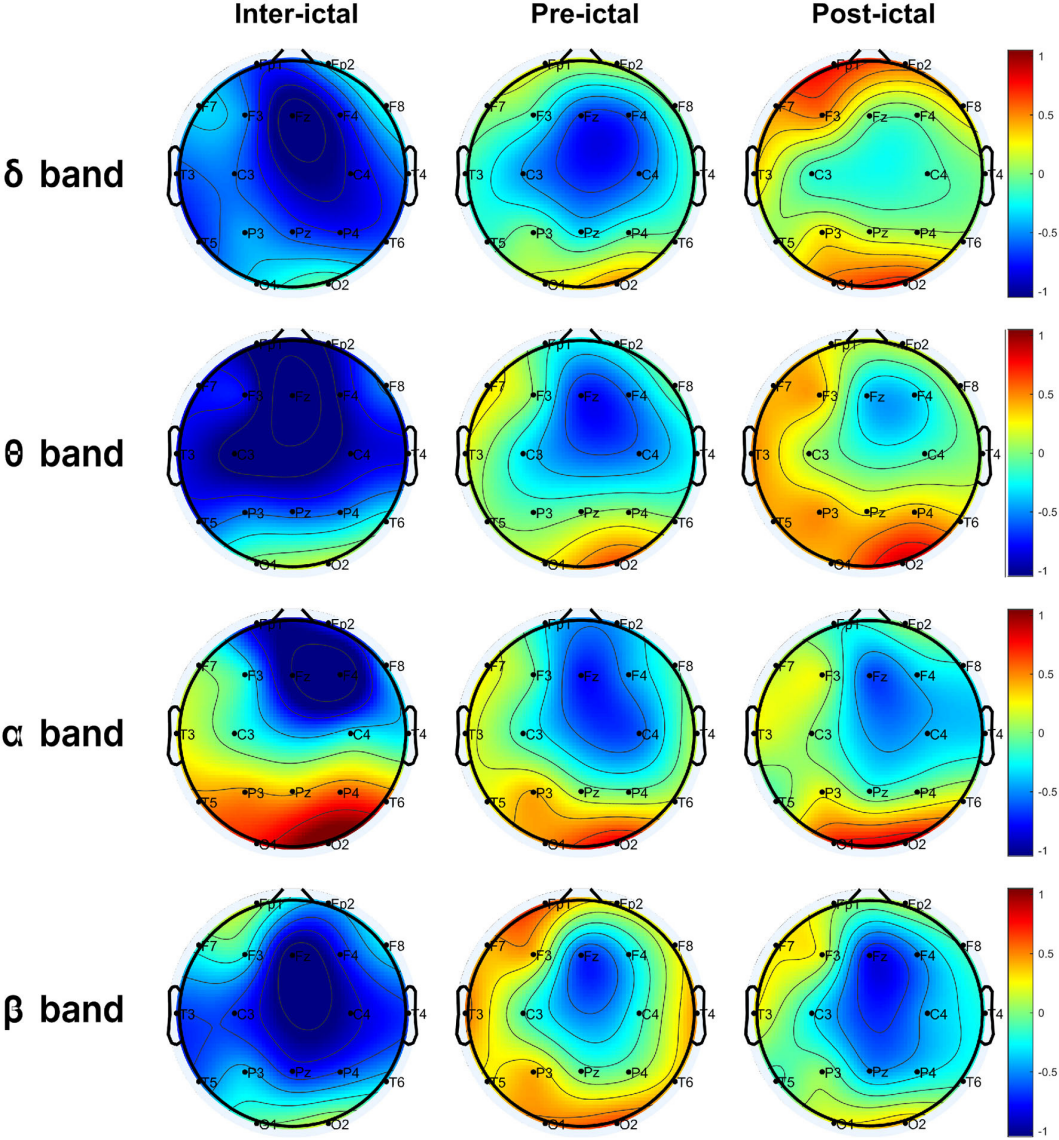
Normalized average energy in the *delta* (δ), *theta* (θ), *alpha* (α), and *beta* (β) bands of all windows corresponding to the interictal, pre-ictal, and post-ictal states.

After calculating the average energies and entropies for each electrode, all features were normalized to Z-score values by subtracting their corresponding mean and dividing by their standard deviation (Figure 3). This approach was chosen to (i) mitigate biases resulting from individual feature scales; and (ii) focus on the nature of the distribution. Subsequently, outliers were removed from each feature. An outlier was defined as a value that fell outside 1.5 times the inter-quartile range below the 1st and above the 3rd quartile. To conduct the statistical analysis based on seizure behavior, the electrodes were categorized into four groups: anterior (Fp1, Fp2, F3, F4, Fz), posterior (O1, O2, P3, P4, Pz), temporal (F7, F8, T7, T8, P5, P6), and central (C3, C4) (Figure 1).

After that, a statistical pipeline was driven on each electrode set. The pipeline consisted of a one-way ANOVA test to evaluate significant differences among the interictal, pre-ictal, and post-ictal intervals for each feature. For the characteristics that showed differences, a Benjamini-Hochberg procedure for multiple comparisons was applied between time intervals. This approach allowed us to identify statistical differences between pairs of ictal windows.

## 3. Results

**Figure 4:**
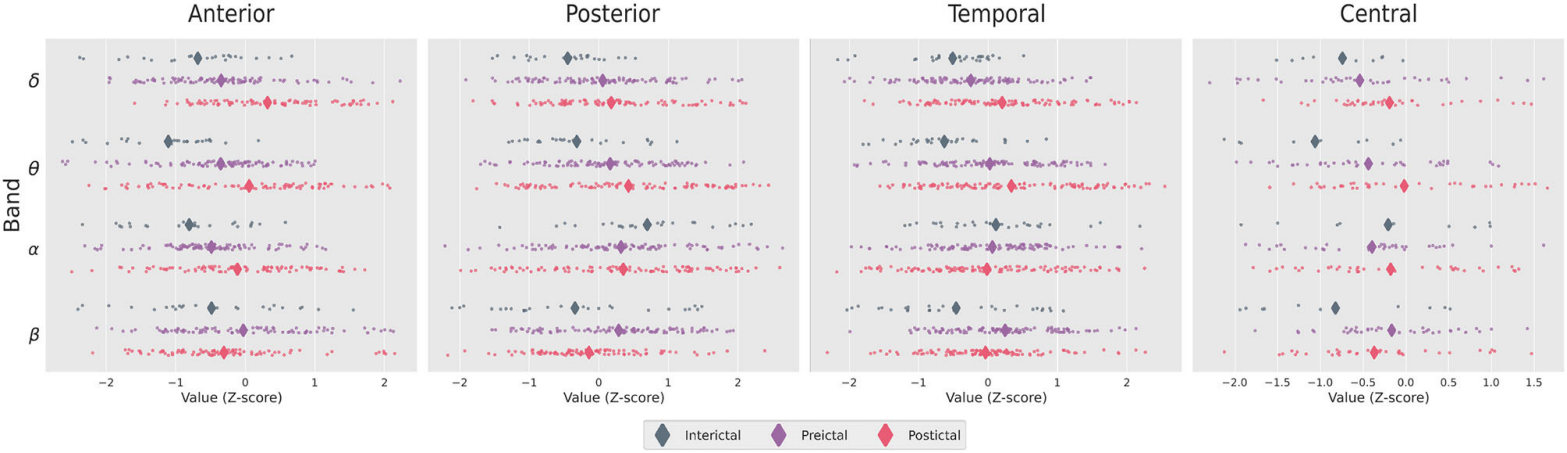
Distribution of Z-score values for the ictal energies in the brain regions studied. Diamonds show the mean of the distribution.

**Figure 5:**
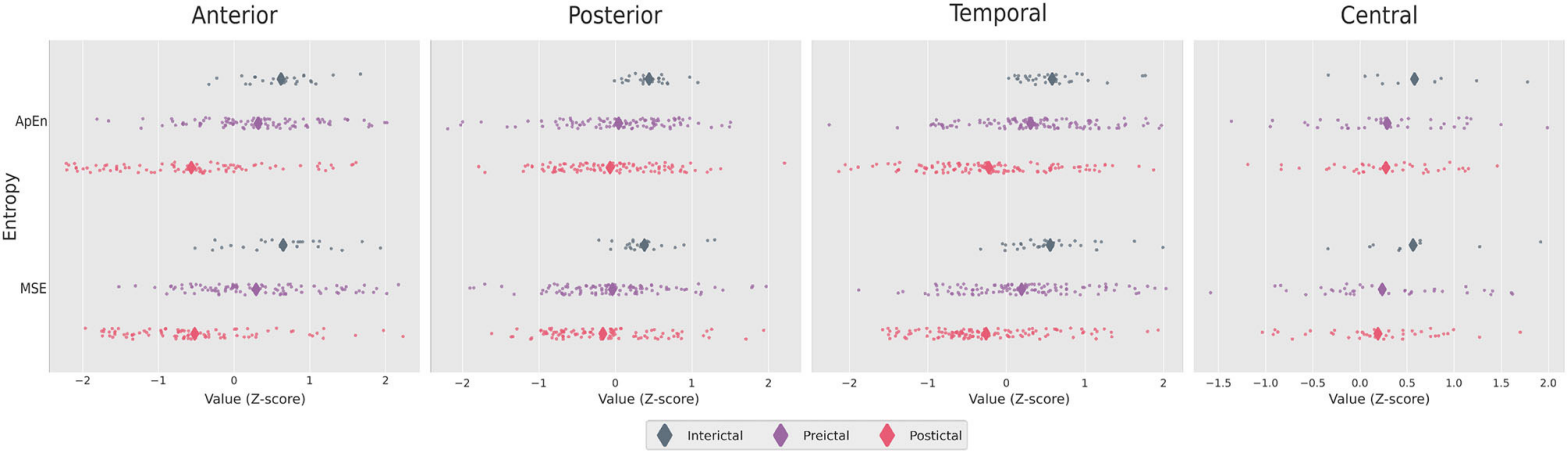
Distribution of Z-score values for the ictal entropies in the brain regions studied. Diamonds show the means of the distributions.

Electrophysiological activity was analyzed during the three intervals identified above: interictal, pre-ictal, and post-ictal, for each frequency band (delta, theta, alpha, beta). The distributions of energy and entropy are presented in Figures 4, 5, respectively. To explore significant differences among these intervals for regional electrodes, a statistical pipeline was performed, as detailed in the Materials and methods section. Results of the statistical analysis are shown in Tables 1, 2.

**Table 1:** *P*-values obtained in the one-way ANOVA tests and their *post-hoc* analyzes for each feature between brain regions.

			**Anterior**		**Posterior**		**Temporal**		**Central**
	Inter-Pre		0.2136		0.0242		0.2667		0.2667
	Inter-Post		0.0000		0.0036		0.0001		NS
*delta*	Pre-Post	0.0000	0.0000	0.0054	0.5760	0.0000	0.0001	NS	NS
	Inter-Pre		0.0016		0.0653		0.0017		0.1089
	Inter-Post		0.0000		0.0018		0.0000		0.0029
*theta*	Pre-Post	0.0000	0.0105	0.0019	0.1305	0.0000	0.0306	0.0026	0.0897
	Inter-Pre		0.2303		NS		NS		NS
	Inter-Post		0.0014		NS		NS		NS
*alpha*	Pre-Post	0.0004	0.0081	NS	NS	NS	NS	NS	NS
	Inter-Pre		NS		0.0055		0.0001		NS
	Inter-Post		NS		0.5773		0.0312		NS
*beta*	Pre-Post	NS	NS	0.0005	0.0029	0.0001	0.0263	NS	NS
	Inter-Pre		0.2757		0.0285		1.0000		NS
	Inter-Post		0.0000		0.0031		0.0000		NS
ApEn	Pre-Post	0.0000	0.0000	0.0048	0.4845	0.0000	0.0000	NS	NS
	Inter-Pre		0.1434		0.0546		0.0491		NS
	Inter-Post		0.0000		0.0015		0.0000		NS
MSE	Pre-Post	0.0000	0.0000	0.0024	0.4144	0.0000	0.0000	NS	NS
	***p*** **-value**	0.001	0.05	0.1	NS				

To facilitate reading and interpretation, red indicates non-significant tests, orange, tests with *p* < 0.1, yellow for *p* < 0.05, and green for *p* < 0.001.

**Table 2:** *P*-values obtained in the one-way ANOVA tests and their *post-hoc* analysis for each feature between brain regions.

			**Inter-ictal**		**Pre-ictal**		**Post-ictal**
	Ant-Temp		NS		0.8421		0.7865
	Ant-Cen		NS		0.6374		0.0141
	Ant-Pos		NS		0.0055		0.7052
	Temp-Cen		NS		0.2645		0.0703
	Temp-Pos		NS		0.0498		0.9971
*delta*	Cen-Pos	NS	NS	0.0005	0.0014	0.0268	0.1169
	Ant-Temp		0.0629		0.0063		0.2493
	Ant-Cen		0.9980		0.9501		0.9841
	Ant-Pos		0.0007		0.0001		0.0867
	Temp-Cen		0.3234		0.0147		0.2817
	Temp-Pos		0.3415		0.6243		0.9232
*theta*	Cen-Pos	0.0006	0.0250	0.0000	0.0008	0.0340	0.1273
	Ant-Temp		0.0013		0.0000		0.8991
	Ant-Cen		0.2646		0.9405		0.9875
	Ant-Pos		0.0000		0.0000		0.0095
	Temp-Cen		0.7402		0.0213		0.8188
	Temp-Pos		0.0678		0.1345		0.0385
*alpha*	Cen-Pos	0.0000	0.0317	0.0000	0.0001	0.0037	0.0299
	Ant-Temp		NS		0.1068		NS
	Ant-Cen		NA		0.8390		NS
	Ant-Pos		NS		0.0569		NS
	Temp-Cen		NS		0.0533		NS
	Temp-Pos		NS		0.9878		NS
*beta*	Cen-Pos	NS	NS	0.0053	0.0307	NS	NS
	Ant-Temp		NS		0.9995		0.0205
	Ant-Cen		NS		0.9967		0.0000
	Ant-Pos		NS		0.0587		0.0003
	Temp-Cen		NS		0.9992		0.0071
	Temp-Pos		NS		0.0626		0.5164
ApEn	Cen-Pos	NS	NS	0.0369	0.3245	0.0000	0.1286
	Ant-Temp		NS		0.8076		0.0803
	Ant-Cen		NS		0.9826		0.0000
	Ant-Pos		NS		0.0165		0.0099
	Temp-Cen		NS		0.9911		0.0105
	Temp-Pos		NS		0.1393		0.8014
MSE	Cen-Pos	NS	NS	0.0207	0.2422	0.0000	0.0791
	***p*** **-value**	0.001	0.05	0.1	NS		

For a more readable interpretation, colors are displayed as red for non-significant tests, orange for those with *p* < 0.1, yellow for *p* < 0.05, and green for *p* < 0.001.

### 3.1. Regions

For the anterior region, we found significant differences in *delta, theta, alpha*, ApEn, and MSE (*p* < 0.001 in all cases). *Post-hoc* analysis revealed that post-ictal *delta* power was higher than interictal (*p* < 0.001) and pre-ictal power (*p* < 0.001), whereas the data for the interictal and pre-ictal periods generated non-significant (NS) differences. Regarding *theta* power, there was a significant difference among the three intervals, with post-ictal showing higher power than pre-ictal and interictal (*p* < 0.05, *p* < 0.001, and *p* < 0.05, respectively). For the *alpha* band, pairwise comparisons showed higher power for the post-ictal compared to the pre-ictal (*p* < 0.05) and interictal (*p* < 0.05). Regarding entropy, both ApEn and MSE were lower for the post-ictal interval compared to the pre-ictal and interictal intervals (*p* < 0.001 in all cases).

In the posterior region, we observed significant differences in *delta, theta*, ApEn and MSE (*p* < 0.05 in all cases) as well as for the *beta* (*p* < 0.001). Pairwise comparisons showed that interictal *delta* power was lower than pre-ictal (*p* < 0.05) and post-ictal (*p* < 0.05), whereas pre-ictal and post-ictal intervals showed non-different powers (NS). Regarding *theta*, only the post-ictal interval showed higher power compared to the interictal one (*p* < 0.05). For the *beta* band, individual comparisons showed lower power for the pre-ictal interval compared to the post-ictal interval (*p* < 0.05), but higher power when compared to the interictal period (*p* < 0.05). Regarding entropy, both ApEn and MSE showed non-significant differences when comparing the pre-ictal and post-ictal intervals (NS).

The temporal region showed a similar pattern to the posterior one, with significant differences in *delta, theta, beta*, ApEn, and MSE (*p* < 0.001 in all cases). *Post-hoc* analysis revealed that post-ictal *delta* power was higher than pre-ictal and interictal (*p* < 0.001 in both cases), while the pre-ictal and interictal intervals showed non-different powers (NS). Regarding *theta*, there was a significant difference among the three intervals, with the post-ictal interval showing higher power than the pre-ictal and interictal periods (*p* < 0.05, *p* < 0.001, and *p* < 0.05, respectively).

For the *beta* band, individual comparisons showed lower power for the pre-ictal interval compared to the post-ictal interval (*p* < 0.05), but higher powet when compared to the interictal period (*p* < 0.001). Regarding entropy, only ApEn showed non-significant difference when comparing the interictal and pre-ictal intervals (NS).

The central region only showed a significant difference between the interictal and post-ictal intervals in *theta* with a higher power in the latter (*p* < 0.05).

### 3.2. Intervals

For the interictal interval, significant differences were observed only for *theta* and *alpha* (*p* < 0.001 in both cases). *Post-hoc* analysis showed that *theta* power was higher in the posterior region that to the anterior and central regions (*p* < 0.001 and *p* < 0.05, respectively). For the *alpha* band, individual comparisons also showed higher power in the posterior region compared to anterior and central regions (*p* < 0.001 and *p* < 0.05, respectively). The temporal region also showed a higher power compared to the anterior one in the *alpha* band (*p* < 0.05).

Regarding the pre-ictal interval, there were differences in *delta, theta, alpha* (*p* < 0.001 in all cases), and *beta*, ApEn and MSE (*p* < 0.05 in these cases). Pairwise comparisons showed that *delta* power was higher in the posterior region compared to the anterior (*p* < 0.05), temporal (*p* < 0.05), and central (*p* < 0.05) regions. Regarding *theta*, there was also a higher power in the posterior region compared to the anterior (*p* < 0.001) and central (*p* < 0.001)regions, but not to the temporal (NS) region. In addition, a higher *theta* power was observed in the temporal region compared to the anterior (*p* < 0.05) and central (*p* < 0.05) regions.

For the *alpha* band, individual comparisons also showed higher power in the posterior region compared to the anterior (*p* < 0.001) and central (*p* < 0.001), but not to the temporal (NS) region. A higher *alpha* power was also observed in the temporal region compared to the anterior (*p* < 0.001) and central (*p* < 0.05) regions. *Beta* power only differed statistically between the posterior and central regions (*p* < 0.05), with a higher power in the former. Regarding entropy, only MSE showed a significant difference between the posterior and anterior regions (*p* < 0.05), with higher entropy in the latter.

With respect to the post-ictal interval, there were differences in *delta* (*p* < 0.05), *theta* (*p* < 0.05), *alpha* (*p* < 0.05), ApEn, and MSE (*p* < 0.001 in both cases). Individual comparisons showed only a higher *delta* power in the anterior compared to the central region (*p* < 0.05). For the *alpha* band, higher power was observed in the posterior region compared to the anterior (*p* < 0.05), temporal (*p* < 0.05), and central (*p* < 0.05) regions. Regarding entropy, these comparisons showed significantly higher ApEn and MSE in the anterior region compared to the temporal (ApEn, *p* < 0.05), central (ApEn and MSE, *p* < 0.001), and posterior (ApEn, *p* < 0.001; MSE, *p* < 0.05) regions. Finally, higher ApEn and MSE values were determined in the temporal region compared to the central region (*p* < 0.05).

## 4. Discussion

Differentiating between ictal and interictal EEG patterns in patients with typical absence seizures has long been a focal point for both clinicians and researchers. Recently, interest has grown in utilizing novel qEEG analysis techniques to further explore these phenomena (Antwi et al., 2019; Li et al., 2022; Springer et al., 2022; Zhong et al., 2022). The primary objective of our study was to investigate whether distinguishable differences exist in the spectral and entropy characteristics of EEGs in the pre-ictal and post-ictal periods compared to the interictal interval in a group of patients with typical absence seizures. By exploring these spectral distinctions. We sought to ascertain whether they can potentially serve as valuable clinical indicators that will aid in differentiating between the ictal and interictal nature of observed epileptiform patterns.

Regarding the slow bands (*delta* and *theta*), we found a significant difference between the interictal and peri-ictal (pre-ictal and post-ictal) intervals across distinct brain regions. This finding partially concurs with previous reports, as some authors have demonstrated an increase in the power of lower bands during the transition from the pre-ictal to ictal, and ictal to post-ictal, states in patients with childhood absence epilepsy (CAE) (Kumar et al., 2021). However, others have reported findings that lead to the opposite conclusion. For example, Li et al. (2022), found a decrease in *delta* oscillations from the interictal to the pre-ictal state in a group of 21 patients with CAE. They proposed that this result could be explained by decreased cortical activity associated with slow oscillation in the brain. We, however, find this explanation difficult to support in light of the corpus of earlier observations.

In clinical practice, visual inspection of background EEG activity in children and adolescents occasionally shows slowing. For example, occipital intermittent rhythmic delta activity (OIRDA) is an interictal pattern commonly seen in CAE patients (Hirsch et al., 2022), some of whom may show a continuous transition from OIRDA to an ictal epileptiform pattern (Aird and Gastaut, 1959; Guilhoto et al., 2006). These authors also suggested that an OIRDA pattern should be interpreted as epileptiform in nature (Guilhoto et al., 2006).

An increase in *delta* and *theta* activity prior to the ictal epileptiform pattern has also been verified in animal models. For example, van Luijtelaar et al. (2011) showed that the simultaneous presence of *delta* and *theta* events in EEGs is a condition for the occurrence of generalized spike-and-wave discharges in WAG/Rij rats. Studies using blood oxygen level dependent functional magnetic resonance imaging (BOLD-fMRI), meanwhile, have evidenced signal increases in various brain regions more than 5s before the onset of electrographic seizures (Bai et al., 2010). It seems, therefore, that a global increase in *delta* and *theta* band power immediately before an ictal epileptiform pattern may be a potential biomarker for differentiation compared to an interictal epileptiform pattern, though this possibility needs to be explored in greater depth.

Our study also showed a significantly different pattern of spectral changes between regions. Although we found an increase in the power of the slow bands across all regions, the most pronounced effect was detected in the posterior area. This finding is not in line with previous work, since some authors have reported a predominant change in the frontal region immediately before the seizure, based on both EEG (Li et al., 2022) and BOLD-fMRI analyzes (Bai et al., 2010).

Several theories on the brain onset of absence seizures have been proposed. Most suggest a complex interplay between cortical (i.e., frontal, parietal) and subcortical (i.e., thalamus) structures (Meeren et al., 2005). Because of the well-known spatial resolution limitation of EEG recordings and our low-density electrode array, we decided not to explore this issue in detail. However, our findings suggest that a quantitative spectral metric with greater posterior focus could be more suitable for differentiating ictal vs. interictal epileptiform patterns in cases of epilepsy with typical absence seizures.

Regarding entropy, our study revealed a significant decrease from the interictal to the pre-ictal and post-ictal intervals, a decrease that had an anterior predominance compared to the other regions. Entropy is a non-linear measure of the uncertainty and has been shown to represent the level of chaos that occurs in the brain (Costa et al., 2002). It can be deduced, then, that a system with higher entropy is more irregular or chaotic. Translating this into brain physiology, there is increasing evidence that various pathological processes are associated with atypical and often, but not always, reduced measures of brain physiological complexity or entropy (Escudero et al., 2006; Bosl et al., 2011). Thus, our findings suggest that EEG-based entropy in anterior regions may be a potential biomarker for differentiating ictal vs. interictal epileptiform patterns in cases of epilepsy marked by typical absence seizures.

## 5. Conclusion

In conclusion, this study focused on investigating distinguishable differences in the spectral characteristics of EEGs during the pre-ictal and post-ictal periods compared to the interictal interval in patients with typical absence seizures. Through the exploration of these spectral distinctions, we sought to identify potential indicators that may aid in differentiating between ictal and interictal epileptiform patterns. As stated above, our findings indicate a significant increase in the power of the slow bands (*delta* and *theta*) during the pre-ictal and post-ictal intervals across several brain regions, especially in the posterior area. This suggests that a global increase in *delta* and *theta* band power immediately before an ictal epileptiform pattern could serve as a potential biomarker for differentiation.

Our study further revealed a significant decrease in entropy from the interictal to the pre-ictal and post-ictal intervals, with a more pronounced effect in the anterior regions. This decrease suggests a reduction in brain complexity during these intervals, and supports the potential use of EEG-based entropy in anterior regions as a biomarker for differentiating epileptiform patterns.

Despite its numerous valuable insights, however, our work has some limitations that must be considered before confidently generalizing its findings. First, we recognize that the sample size was relatively small, though our research marks a crucial initial step in quantitatively addressing this issue in clinical practice. As we continue forward, expanding the sample size will undoubtedly strengthen the robustness of our conclusions. Second, the absence of a control group (a set of interictal epileptiform patterns) might initially limit the clinical application of our findings. Nonetheless, we are committed to performing a second-step validation study to validate and refine our results, to ensure their reliability and usefulness for the wider medical community. Third, while we explored specific quantitative metrics in this study, we are aware of the potential of other, more complex quantitative measures, for clinical practice, as other researchers have demonstrated (Kramer and Kromm, 2019; Munjal et al., 2022). We envision incorporating these advanced techniques into our future research endeavors to expand the depth and breadth of our findings.

Despite these limitations, our findings support the assertion that quantitative analysis of pre-ictal and post-ictal intervals can be potential EEG biomarkers to identify the ictal nature of the epileptiform discharge and potentially differentiating it from the interictal one in patients with confirmed or suspected typical absence seizures. Future research, with larger sample sizes and more complex quantitative methods, is warranted to validate and expand these findings.

## Data availability statement

The data that support the findings of this study are available from the corresponding authors, RR-V and HV-P, upon reasonable request.

## Ethics statement

The study protocol was approved by the Ethics Committee of the Country 2000 Hospital. The studies were conducted in accordance with the local legislation and institutional requirements. The participants provided their written informed consent to participate in this study. Written informed consent was obtained from the individual(s) for the publication of any potentially identifiable images or data included in this article.

## Author contributions

AG-A: Writing—original draft, Conceptualization, Data curation, Funding acquisition, Investigation, Methodology, Project administration, Resources, Supervision, Validation, Writing—review and editing. ER-P: Investigation, Writing—original draft, Writing—review and editing, Formal analysis, Software, Visualization. OR-Q: Investigation, Writing—original draft, Writing—review and editing, Formal analysis, Software, Visualization. OP: Formal analysis, Investigation, Methodology, Software, Validation, Visualization, Writing—original draft, Writing—review and editing. EG-Q: Software, Visualization, Writing—original draft. AG-E: Data curation, Validation, Resources, Writing—original draft. RR-V: Conceptualization, Funding acquisition, Investigation, Methodology, Project administration, Resources, Supervision, Validation, Writing—original draft, Writing—review and editing. HV-P: Conceptualization, Funding acquisition, Investigation, Methodology, Project administration, Resources, Supervision, Validation, Writing—original draft, Writing—review and editing.
